# Potential Influence of Anesthetic Interventions on Breast Cancer Early Recurrence According to Estrogen Receptor Expression: A Sub-Study of a Randomized Trial

**DOI:** 10.3389/fonc.2022.837959

**Published:** 2022-02-10

**Authors:** Mohan Li, Yuelun Zhang, Lijian Pei, Zhiyong Zhang, Gang Tan, Yuguang Huang

**Affiliations:** ^1^Department of Anesthesiology, Peking Union Medical College Hospital, Chinese Academy of Medical Sciences & Peking Union Medical College, Beijing, China; ^2^Medical Research Center, Peking Union Medical College Hospital, Chinese Academy of Medical Sciences and Peking Union Medical College, Beijing, China; ^3^Outcomes Research Consortium, Cleveland, OH, United States

**Keywords:** anesthesia, breast cancer, estrogen receptor, recurrence, propofol

## Abstract

**Background:**

Effects of anesthetic interventions on cancer prognosis remain controversial. There is evidence that estrogen receptor (ER)-negative breast cancer patients have an early recurrence peak. We aimed to assess the potential benefit of regional anesthesia-analgesia versus general anesthesia regarding early recurrence in breast cancer according to ER expression.

**Methods:**

Based on a multicenter randomized controlled trial (clinicaltrials.gov, NCT00418457), we included all the patients from Peking Union Medical College Hospital research center in this study. The primary outcome was breast cancer recurrence after surgery. The Cox proportional hazard model was used to compare recurrence between groups.

**Results:**

In total, 1,253 breast cancer patients were included in this sub-study, among whom the median follow-up time was 53 months. In this sub-study, 320 patients were ER-negative, and 933 were ER-positive. As for ER-negative patients, the recurrence risk in the PPA (paravertebral blocks and propofol general anesthesia) group showed no statistical difference compared with the GA (sevoflurane and opioids general anesthesia) group (19.1% versus 23.4%; adjusted HR: 0.80, 95% CI: 0.50–1.30; *P* = 0.377). In the first 18 months after breast cancer surgery, which is considered as the classical early peak of recurrence, after adjustment for menstruation and the pathological stage of tumor, the decrease of early recurrence observed in the PPA group was not significant compared with the GA group (adjusted HR: 0.63, 95% CI: 0.34–1.14; *P* = 0.127).

**Conclusions:**

In our study, the effects of early recurrence after breast cancer surgery in both ER-negative and ER-positive patients were similar between regional anesthesia-analgesia and general anesthesia. Large samples of ER-negative patients will be needed to clarify the effects of anesthetic interventions.

## Introduction

Breast cancer is the most commonly diagnosed cancer type and is also the leading cause of cancer death in women (1). Despite the evolving process of treatment methods, breast cancer recurrence remains a major problem that affects patient prognosis. Cell phenotype affects recurrence. The annual hazard rate of recurrence in estrogen receptor (ER)-positive patients is higher beyond 5 years than that in ER-negative patients. Conversely, during the first 5 years, the annual hazard rates of recurrence are higher in ER-negative patients (2). An early peak of recurrence could be seen classically in the first 18 months after breast cancer surgery (3, 4), which is in accordance with the annual hazard rate peak observed in ER-negative breast cancer around years two and three after initial diagnosis (5).

Various research demonstrated that the surgical stress response may increase the risk of breast cancer dissemination and metastasis during and after surgery (6). Regional anesthesia-analgesia is thought to prevent cancer recurrence by influencing angiogenesis, moderating the neuroendocrine system, and affecting immunity (7). Moreover, some studies indicate that compared to sevoflurane, propofol attenuates the inflammatory response, which may finally reduce the risk of cancer recurrence (8). However, these findings were only observed in animal studies and retrospective clinical research (9–11), but not in prospective clinical trials (12, 13), which makes the relationship between anesthetic interventions and breast cancer recurrence controversial. It is worth noting that ER-negative breast cancer cells were usually used to explore the relationship between anesthesia and cancer recurrence in both *in vivo* and *in vitro* studies, but in clinical studies, the subtypes of breast cancer were rarely considered. Only few clinical trials were focused on different breast cancer subtypes (14, 15).

Previous studies indicate that the early recurrence peak of breast cancer may be resulted from dormant cell division and angiogenesis induced by operation (16), while the late peak is considered to be the result of metastasis dormancy and which is most common in ER-positive subtype (4). Compared to ER-positive breast cancer cells, ER-negative cells tend to be associated with more invasion and more related to early recurrence (17). Biological studies showed that anesthetic interventions may influence breast cancer early recurrence. It can increase cell apoptosis and reduce proliferation (18, 19) and alter the angiogenesis factors and cancer immunomodulatory cytokines in serum, thereby affecting the functions of ER-negative breast cancer cells (20, 21). Furthermore, the decrease of methylation can reactivate suppressor genes and lead to the inhibition of cancer (22). Ropivacaine could decrease methylation in ER-negative cells rather than ER-positive cells (23). Therefore, it is logically reasonable to hypothesize that ER-negative patients could benefit more from regional anesthesia-analgesia especially in early recurrence.

Current clinical studies have seldom reported the effects of anesthetic interventions in specific cancer cell phenotypes. Considering the gap of current research, we tried to test whether patients according to ER expression status would have an increased benefit on early recurrence from regional anesthesia-analgesia compared with general anesthesia.

## Methods

### Patients

This study was based on a previous multicenter randomized controlled trial (clinicaltrials.gov, NCT00418457) (12). The Peking Union Medical College Hospital (PUMCH) ethics committee approved the protocol of the original randomized controlled trial (S-638) on January 23^rd^, 2014, and all patients understood and signed informed consent for participation in the previous study. Patients receiving primary breast cancer surgery at PUMCH who met the following inclusion criteria were enrolled: aged 18–85 years, and American Society of Anesthesiologists physical status I–III. Patients with contraindications for either anesthetic approach were excluded.

### Anesthetic Interventions

Patients were randomly assigned at a 1:1 ratio *via* a computer-generated random sequence to regional anesthesia-analgesia (PPA) group or general anesthesia (GA) group, and received either paravertebral blocks and propofol or sevoflurane and opioids respectively (24). Thirty minutes prior to the induction of anesthesia, patients of the PPA group underwent a single thoracic paravertebral nerve block under ultrasound guidance, using a multipoint method (T1~T5) to inject 5 ml 0.75% ropivacaine at each puncture point. Patients of the GA group were positioned in a similar manner as those of the PPA group, while received 0.2 ml 1% lidocaine injections at each puncture point for local infiltration anesthesia only. Analgesia in the PPA group was primarily based on paravertebral blocks, and maintained using propofol target-controlled infusion (effect site concentration: 2.5–4.0 μg/ml, Marsh model). In the GA group, general anesthesia was induced with 2 mg/kg propofol, and maintained with 2% sevoflurane. Both groups received 1–2 μg/kg fentanyl and 0.4–0.6 mg/kg rocuronium at the induction of anesthesia to facilitate laryngeal mask insertion. During each patient’s operation, additional intravenous fentanyl and rocuronium were provided intermittently, and blood pressure and heart rate within a 20% range of basic values were maintained (25).

### Outcome

The primary outcome was breast cancer recurrence, which was assessed by contacting patients or the specialist every 6 months. Time to recurrence was measured from the date of surgery to the earliest date that recurrence was detected at any site. Clinical evidence such as radiographic examinations or pathologic findings was provided to confirm recurrence. Medical records were provided including demographic characteristics, clinical factors, and pathological factors related to breast cancer recurrence.

### Statistical Analysis

The analysis was performed by the intention-to-treatment principle. Patients lost to follow-up were censored at the time of last contact. To assess the validity of our hypothesis, that there is an interaction between anesthetic interventions and cell phenotype, the data analysis was performed based on ER status. Breast cancer recurrence rates were analyzed using Poisson regression and Cox proportional hazard models, and were adjusted for confounders. Prognostic factors for recurrence that were unequally distributed among intervention groups in the PUMCH population were regarded as confounders. A standardized mean difference was used to assess distributions of prognostic factors among groups, and a threshold of < 0.1 was considered a negligible difference (26). In addition to confounders identified using the standardized mean difference, other factors were considered clinically important for breast cancer recurrence. Hence, the following two models were devised: Model 1, which was created by adjusting for confounders that were unequally distributed between the two groups; and Model 2, which considered predetermined factors including age, tumor-node-metastasis (TNM) stage, nuclear grade, postoperative radiotherapy, and chemotherapy.

The proportional hazard assumption was tested by evaluating the statistical significance of the anesthesia group-by-time interaction. Because the classical early peak of recurrence is usually observed 18 months post-surgery, we used a split function to explore time-varying coefficients using 18 months as a prespecified cut-off time point (27). Different time splitting points were tested *via* sensitivity analyses. One patient died before recurrence. Therefore, a competing risk analysis was conducted.

This was a sub-study of a randomized controlled trial, which included patients from a single study site. Therefore, the sample size was predetermined. We estimated the statistical power of the study using the available sample size. In the sub-study, recurrence was observed in 68 patients of the ER-negative population. Our sub-study was able to detect a 20% reduction in ER-negative breast cancer recurrence using an event-driven design with a statistical power of 15%.

All statistical tests were two-sided. The significance level was set at 0.05. Statistical analyses were performed using R version 4.0.2 (R Foundation for Statistical Computing, Vienna, Austria; URL: https://www.R-project.org/.) with “cmprsk”, “gsDesign”, “prodlim”, “stats”, “survival”, “survminer”, “tableone” and “tidyverse” packages. Plots were created using GraphPad PRISM 8.2.0 (GraphPad Software company, San Diego, California, USA; URL: https://www.graphpad.com/scientific-software/prism/).

## Results

From February 8^th^, 2014, to December 8^th^, 2016, 1,253 patients from PUMCH research center were included in this sub-study. Patients were followed-up till December 8^th^, 2019, except for 11 patients who were lost to follow-up, patients either reported recurrence or completed at least 3 years of follow-up. The median follow-up time was 53 (IQR 44-62) months. In total, 624 patients were assigned to the PPA group, and 629 patients were assigned to the GA group. Some exposures were unequally distributed between the two groups, and were therefore considered as potential confounders when the association between anesthesia method and cancer recurrence was assessed. These included menstruation status and pathological stage of tumor (**Supplementary Table 1**). Since recurrence was infrequently observed in T0 and TNM stage 0 patients, pathological stage of tumor and tumor TNM stage were regrouped as binary variables.

When the full dataset of the PUMCH population was considered, anesthetic interventions did not affect recurrence after breast cancer surgery (unadjusted hazard ratio [HR]: 0.96, 95% confidence interval [CI]: 0.71–1.30; *P* = 0.778). In Model 1, menstruation and pathological stage of tumor were considered confounders of the multivariable Cox regression, and the HR of regional anesthesia-analgesia compared with general anesthesia was 0.92 (95% CI: 0.68–1.26; *P* = 0.612). After adjusting for predetermined factors (Model 2) including age, tumor TNM stage, nuclear grade, postoperative chemotherapy and radiotherapy, the HR of regional anesthesia-analgesia was 0.92 (95% CI: 0.67–1.26; *P* = 0.598).

**Figure 1:**
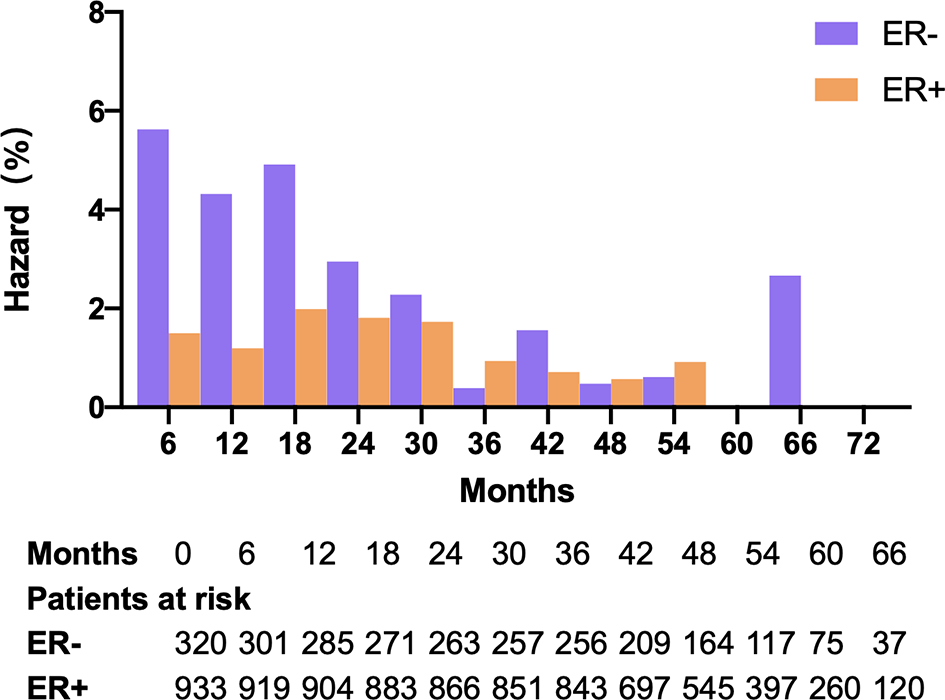
Hazard rate per 6 months in ER-negative patients and ER-positive patients.

In this sub-study, 320 and 933 patients were ER-negative and -positive, respectively, based on pathological results (**Table 1**). We observed a peak in early recurrence in ER-negative patients (**Figure 1**). Recurrence risk was higher in ER-negative patients than ER-positive patients (21.3% versus 10.4%, respectively; adjusted relative risk [RR]: 1.91; 95% CI: 1.39-2.61; *P* < 0.001). Further analyses were conducted in ER-negative and ER-positive subgroups separately.

**Table 1:** Demographic and clinical characteristics according to ER status.

	ER status = negative (n = 320)		ER status = positive (n = 933)	
	PPA	GA	PPA	GA
	(n = 162)	(n = 158)	(n = 462)	(n = 471)
**Demographics**				
Age, yr	50 ± 10	48 ± 10	48 ± 10	49 ± 9
Menstruation, n (%)				
Premenopausal	58 (35.8)	61 (38.6)	221 (47.8)	215 (45.6)
Perimenopausal	11 (6.8)	21 (13.3)	52 (11.3)	63 (13.4)
Postmenopausal	93 (57.4)	76 (48.1)	189 (40.9)	193 (41.0)
Body mass index, kg/m^2^	23.9 ± 3.3	23.9 ± 3.6	23.6 ± 3.1	23.7 ± 3.3
ASA physical status, n (%)				
I	113 (69.8)	111 (70.3)	334 (72.3)	318 (67.5)
II	49 (30.2)	47 (29.7)	127 (27.5)	151 (32.1)
III	0 (0.0)	0 (0.0)	1 (0.2)	2 (0.4)
Neoadjuvant, n (%)	9 (5.6)	8 (5.1)	16 (3.5)	14 (3.0)
**Primary tumor**				
Tumor side, n (%)				
Left	89 (54.9)	79 (50.0)	225 (48.7)	236 (50.1)
Right	71 (43.8)	77 (48.7)	233 (50.4)	224 (47.6)
Bilateral	2 (1.2)	2 (1.3)	4 (0.9)	11 (2.3)
Nuclear grade, n (%)				
1/2	57 (38.3)	47 (30.9)	328 (76.3)	334 (76.8)
3	92 (61.7)	105 (69.1)	102 (23.7)	101 (23.2)
Unknown	13 (8.0)	6 (3.8)	32 (6.9)	36 (7.6)
PR status, n (%)				
Negative	146 (90.1)	148 (93.7)	49 (10.6)	63 (13.4)
Positive	16 (9.9)	10 (6.3)	413 (89.4)	407 (86.6)
Unknown	0 (0.0)	0 (0.0)	0 (0.0)	1 (0.2)
HER2 status, n (%)				
Negative	72 (44.4)	69 (43.7)	299 (64.7)	321 (68.2)
Positive	84 (51.9)	84 (53.2)	115 (24.9)	104 (22.1)
Equivocal	6 (3.7)	5 (3.2)	48 (10.4)	46 (9.8)
Pathology stage, tumor (T), n (%)				
T0 or Tis	10 (6.2)	6 (3.9)	12 (2.6)	14 (3.0)
T1	70 (43.5)	73 (47.1)	262 (57.1)	293 (62.3)
T2	74 (46.0)	61 (39.4)	172 (37.5)	151 (32.1)
T3	7 (4.3)	12 (7.7)	12 (2.6)	11 (2.3)
T4	0 (0.0)	3 (1.9)	1 (0.2)	1 (0.2)
Pathology stage, nodes (N), n (%)				
N0	92 (56.8)	84 (53.2)	249 (53.9)	261 (55.5)
N1	37 (22.8)	24 (15.2)	111 (24.0)	129 (27.4)
N2	16 (9.9)	19 (12.0)	46 (10.0)	42 (8.9)
N3	17 (10.5)	31 (19.6)	56 (12.1)	38 (8.1)
Tumor TNM stage, n (%)				
0	9 (5.6)	5 (3.2)	9 (2.0)	14 (3.0)
1	43 (26.7)	47 (29.7)	169 (36.8)	190 (40.5)
2	74 (46.0)	54 (34.2)	175 (38.1)	184 (39.2)
3	35 (21.7)	52 (32.9)	106 (23.1)	81 (17.3)
**Intraoperative**				
Surgery type, n (%)				
Simple mastectomy	27 (16.7)	21 (13.3)	49 (10.6)	66 (14.0)
Modified radical	120 (74.1)	109 (69.0)	324 (70.1)	324 (68.8)
Wide local excision with node dissection	9 (5.6)	23 (14.6)	59 (12.8)	50 (10.6)
Others	6 (3.7)	5 (3.2)	30 (6.5)	31 (6.6)
Drugs				
Propofol, mg	531 [434, 677]	120 [100, 130]	502 [430, 650]	120 [100, 130]
Fentanyl, μg	100 [50, 100]	200 [185, 250]	100 [50, 100]	200 [190, 250]
Lidocaine, mg	20 [0, 40]	40 [0, 40]	20 [0, 40]	30 [0, 40]
NSAIDS, n (%)	6 (3.7)	6 (3.8)	10 (2.2)	6 (1.3)
**Postoperative treatment**				
Radiotherapy, n (%)	55 (34.0)	73 (46.2)	200 (43.3)	164 (34.8)
Chemotherapy, n (%)	148 (91.4)	144 (91.1)	336 (72.7)	322 (68.4)
Endocrine therapy, n (%)	16 (9.9)	13 (8.2)	393 (85.1)	399 (84.7)
Herceptin, n (%)	46 (28.4)	47 (29.7)	73 (15.8)	59 (12.5)
Recurrence, n (%)	31 (19.1)	37 (23.4)	50 (10.8)	47 (10.0)

Results presented as $\bar{x}$ ± s or median (P_25_, P_75_) or n (%).

ER, estrogen receptor; PPA, paravertebral block with propofol general anesthesia; GA, fentanyl with sevoflurane general anesthesia; ASA, American Society of Anesthesiologist; PR, progesterone receptor; HER2, human epidermal growth factor receptor 2.

**Figure 2:**
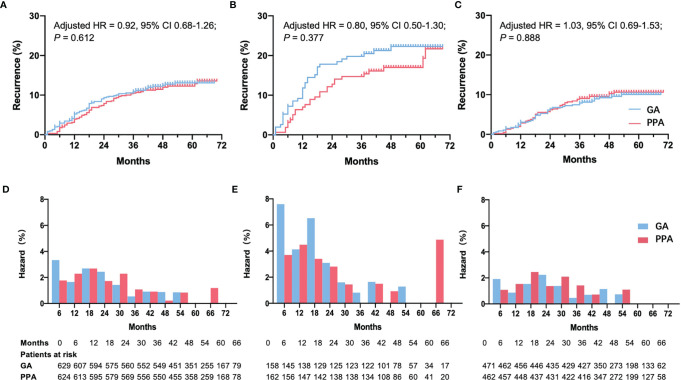
Recurrence curve and hazard rate per 6 months among patients who were given PPA or GA. Recurrence curve in all patients **(A)**, ER-negative patients **(B)**, and ER-positive patients **(C)**. Hazard rate per 6 months in all patients **(D)**, ER-negative patients **(E)**, and ER-positive patients **(F)**. ER, estrogen receptor; PPA, paravertebral block with propofol general anesthesia; GA, fentanyl with sevoflurane general anesthesia; HR, hazard ratio; CI, confidence interval. HR, 95% CI and *P* values were reported using adjusted multivariable Cox regression Model 1 adjusted for menstruation and pathology stage of tumor (binary).

158 ER-negative patients were placed in the GA group, and 162 were placed in the PPA group. Recurrence risk among those in the PPA group was not reduced versus the GA group (19.1% versus 23.4%, respectively; adjusted RR: 0.86, 95% CI: 0.53–1.39; *P* = 0.542), and the adjusted HR was 0.80 (95% CI: 0.50–1.30; *P* = 0.377) (**Figures 2A, D**). No violation of the proportional hazard assumption was observed (*P* = 0.122). To assess the potential benefit of regional anesthesia-analgesia on early recurrence in ER-negative patients, a step function with a predefined time splitting point of 18 months was used to perform an extended Cox regression analysis. Throughout a period of < 18 months after surgery, the unadjusted HR for regional anesthesia-analgesia was 0.62 (95% CI: 0.34–1.13; *P* = 0.122). After adjusting for confounders using Model 1, the HR for early recurrence was 0.63 (95% CI: 0.34–1.14; *P* = 0.127). For periods exceeding the classical recurrence peak of 18 months, the effect of regional anesthesia seemed limited (adjusted HR: 1.33, 95% CI: 0.57–3.12; *P* = 0.513).

The same result was obtained using Model 2. Although it was not statistically significant (**Table 2**), the incidence curve revealed that anesthetic interventions may influence rates of early recurrence in ER-negative patients (**Figures 2B, E**). We also tested other splitting points *via* sensitivity analyses, since the early peak in recurrence was reported to occur between the first and third year (5); however, results were unaffected. The multivariable Cox regression model satisfied the proportional hazard assumption in the ER-positive group (*P* = 0.859). Both models showed that anesthetic interventions did not significantly affect recurrence (Model 1: adjusted HR, 1.03; 95% CI 0.69–1.53; *P* = 0.888; Model 2: adjusted HR, 1.06; 95% CI, 0.71–1.60; *P* = 0.764). The recurrence curve of ER-positive group was not affected by anesthetic interventions (**Figures 2C, F**).

**Table 2:** Extended Cox regression model with estimated recurrence hazard ratios of PPA *vs.* GA in ER-negative group.

Variables	HR (95% CI)	*P*
Unadjusted Model^a^		
PPA *vs.* GA	0.77 (0.48-1.25)	0.290
PPA *vs.* GA (T ≤ 18m)^b^	0.62 (0.34-1.13)	0.122
PPA *vs.* GA(T>18m)^b^	1.16 (0.51-2.66)	0.717
Model 1		
PPA *vs.* GA	0.80 (0.50-1.30)	0.377
PPA *vs.* GA(T ≤ 18m)^b^	0.63 (0.34-1.14)	0.127
PPA *vs.* GA(T>18m)^b^	1.33 (0.57-3.12)	0.513
Model 2		
PPA *vs.* GA	0.82 (0.49-1.36)	0.438
PPA *vs.* GA(T ≤ 18m)^b^	0.72 (0.39-1.34)	0.309
PPA *vs.* GA(T>18m)^b^	1.05 (0.44-2.54)	0.908

Model 1 was adjusted for menstruation and pathology stage of tumor (binary) using multivariable extended Cox regression.

Model 2 was adjusted for age, Tumor TNM stage (binary), nuclear grade, postoperative radiotherapy and postoperative chemotherapy using multivariable extended Cox regression.

PPA, paravertebral block with propofol general anesthesia; GA, fentanyl with sevoflurane general anesthesia; HR, hazard ratio; CI, confidence interval.

a Analyzed with univariable Cox regression model.

b Analyzed with step function model.

One death in an ER-negative patient occurred due to heart disease prior to cancer recurrence. The competing risk model showed that the difference of recurrence was not significant in ER-negative patients between PPA and GA group (Model 1: adjusted HR, 0.81; 95% CI, 0.50–1.31; *P* = 0.390; Model 2: adjusted HR, 0.83; 95% CI, 0.50–1.39; *P* = 0.480).

## Discussion

Several studies have investigated the effects of anesthetic interventions on cancer prognosis in recent years (10, 28–34). Although some *in vitro* studies and observational analyses have reported beneficial effects of regional anesthesia on cancer recurrence, most randomized studies have shown that regional anesthesia does not improve breast cancer recurrence-free survival. In our study, similar to findings of the original multicenter randomized trial (12), regional anesthesia-analgesia showed no statistical difference in risk of early recurrence after breast cancer surgery in the ER-negative population. However, an early recurrence peak was clearly observed among the ER-negative group, and the trend of reduced risk could also be seen in the ER-negative patients rather than ER-positive patients under regional anesthesia-analgesia.

Although the use of regional anesthesia-analgesia for breast cancer surgery minimizes the alteration of cytokines and inflammation, and improves the immune response, there is no convincing clinical evidence that supports or refutes the clinical use of regional anesthesia-analgesia to reduce the risk of cancer recurrence (35–37). One possible reason why results of *in vitro* studies cannot be reproduced in clinical trials is that cells are subjected to prolonged exposure to local anesthetics for 72 hours *in vitro* (23), while short-term exposure of anesthetics in the clinical context seems negligible. Another reason for the discrepancy may be related to the fact that trauma due to breast cancer surgery is relatively less significant than that of other types of surgeries (38, 39). However, a range of randomized trials focusing on major surgeries have also revealed a similar effect in reducing recurrence due to the administration of regional anesthesia-analgesia (28, 29). More importantly, intrinsic biological characteristics of tumors and treatments can also affect recurrence besides anesthetic interventions (40).

However, although there is no statistically significant difference in the risks of recurrence between PPA and GA groups when assessed according to ER expression status, a trend of reduced recurrence hazard could be observed under regional anesthesia-analgesia during the first 18 months in the ER-negative patients in our study, but not in ER-positive patients. Breast cancer cell phenotypes have distinct biological behaviors, with differing recurrence curves (5), of which ER-negative patients rather than ER-positive patients have a higher early recurrence peak (2), consistent with our results. The early recurrence peak is often interpreted as a break in dormancy, which is induced by growth stimulating factors after surgery (41), and may be reduced by regional anesthesia due to its immune-preserving and anti-inflammatory qualities (3, 42). The effects of anesthetic interventions on cancer recurrence may vary among different cytotypes (43). Lirk P et al. found that the demethylation effect of local anesthetics is more significant in ER-negative cells compared with ER-positive cells, leading to the inhibition of cancer. As a result, impacts of regional anesthesia-analgesia on cancer recurrence may be greater in ER-negative patients than ER-positive patients.

A strength of this randomized study is the large proportion of patients who were successfully followed up. Secondly, the patients were from the same study center, so that to circumvent bias with a similar genetic background. However, it does have some limitations. Most importantly, the sample size of ER-negative patients may not have been large enough to assess the influence of regional anesthesia-analgesia on recurrence. In the original multicenter randomized controlled trial, the sample size was selected to assess a 30% reduction in cancer recurrence. However, a recent meta-analysis focusing on late-stage patients revealed only a slight benefit from regional anesthesia use, decreasing cancer recurrence by 4%–12% (32). Similarly, a nationwide retrospective cohort study also revealed a slight decrease of total intravenous anesthesia compared with volatile anesthesia in recurrence-free survival (2%–13%) (34). Although regional anesthesia-analgesia seemed to benefit ER-negative patients in this study with a decrease of 20% in cancer recurrence, far more ER-negative patients should be considered. Also, this study included two factors of regional anesthesia vs. general anesthesia, and propofol intravenous anesthesia vs. sevoflurane inhalation anesthesia simultaneously. As a result, the effect is combined and difficult to identify if it is regional anesthesia or propofol has any potential influence on the early recurrence of ER-negative breast cancer patients.

In summary, rates of early recurrence in both ER-negative and ER-positive breast cancer were similar between regional anesthesia-analgesia using paravertebral blocks and propofol and general anesthesia by sevoflurane and opioids. However, the recurrence curve revealed a potential benefit of regional anesthesia-analgesia in ER-negative patients. Large samples of high-risk patients (such as ER-negative patients) will be needed to clarify the influence of anesthetic interventions.

## Data Availability Statement

The datasets presented in this article are not readily available because the datasets were obtained from the hospital information system, which is not openly accessible to other individuals or institutions. Requests to access the datasets should be directed to Lijian Pei, hazelbeijing@vip.163.com.

## Ethics Statement

The studies involving human participants were reviewed and approved by Peking Union Medical College Hospital ethics committee (S-638). The patients/participants provided their written informed consent to participate in this study.

## Author Contributions

LP and YH contributed to the study design. ML, ZZ, GT, and LP contributed to the data acquisition. ML and YZ contributed to the data analysis. ML and LP wrote the report. All authors reviewed the report and approved it for publication.

## Funding

This study was supported by Peking Union Medical College Hospital Precipitation and Integration Foundation (ZC201906511).

## Conflict of Interest

The authors declare that the research was conducted in the absence of any commercial or financial relationships that could be construed as a potential conflict of interest.

## Publisher’s Note

All claims expressed in this article are solely those of the authors and do not necessarily represent those of their affiliated organizations, or those of the publisher, the editors and the reviewers. Any product that may be evaluated in this article, or claim that may be made by its manufacturer, is not guaranteed or endorsed by the publisher.
