# Supplementary material for: Potential Influence of Anesthetic Interventions on Breast Cancer Early Recurrence According to Estrogen Receptor Expression: A Sub-Study of a Randomized Trial
Source: Front Oncol. 2022 Feb 10;12:837959. doi: 10.3389/fonc.2022.837959 (PMC8869606; doi:10.3389/fonc.2022.837959)
Supplement: Supplementary file 1 [file Table_1.docx]

Supplement Table 1 Demographic and clinical characteristics of participants

|  | Total  (n = 1253) | PPA  (n = 624) | GA  (n = 629) | SMD |
| --- | --- | --- | --- | --- |
| **Demographics** |  |  |  |  |
| Age, yr | 49 ± 10 | 49 ± 10 | 49 ± 9 | 0.001 |
| Menstruation, n (%) |  |  |  | 0.103 |
| Premenopausal | 555 (44.3) | 279 (44.7) | 276 (43.9) |  |
| Perimenopausal | 147 (11.7) | 63 (10.1) | 84 (13.4) |  |
| Postmenopausal | 551 (44.0) | 282 (45.2) | 269 (42.8) |  |
| Body mass index, kg/m^2^ | 23.7 ± 3.3 | 23.7 ± 3.2 | 23.8 ± 3.3 | 0.025 |
| ASA physical status, n (%) |  |  |  | 0.080 |
| Ⅰ | 876 (69.9) | 447 (71.6) | 429 (68.2) |  |
| Ⅱ | 374 (29.8) | 176 (28.2) | 198 (31.5) |  |
| Ⅲ | 3 (0.2) | 1 (0.2) | 2 (0.3) |  |
| Neoadjuvant, n (%) | 47 (3.8) | 25 (4.0) | 22 (3.5) | 0.027 |
| **Primary tumor** |  |  |  |  |
| Tumor side, n (%) |  |  |  | 0.091 |
| Left | 629 (50.2) | 314 (50.3) | 315 (50.1) |  |
| Right | 605 (48.3) | 304 (48.7) | 301 (47.9) |  |
| Bilateral | 19 (1.5) | 6 (1.0) | 13 (2.1) |  |
| Nuclear grade, n (%) |  |  |  | 0.033 |
| 1/2 | 766 (65.7) | 385 (66.5) | 381 (64.9) |  |
| 3 | 400 (34.3) | 194 (33.5) | 206 (35.1) |  |
| Unknown | 87 (6.9) | 45 (7.2) | 42 (6.7) |  |
| ER status, n (%) |  |  |  | 0.019 |
| Negative | 320 (25.5) | 162 (26.0) | 158 (25.1) |  |
| Positive | 933 (74.5) | 462 (74.0) | 471 (74.9) |  |
| PR status, n (%) |  |  |  | 0.050 |
| Negative | 406 (32.4) | 195 (31.3) | 211 (33.6) |  |
| Positive | 846 (67.6) | 429 (68.8) | 417 (66.4) |  |
| Unknown | 1 (0.1) | 0 (0.0) | 1 (0.2) |  |
| HER2 status, n (%) |  |  |  | 0.052 |
| Negative | 761 (60.7) | 371 (59.5) | 390(62.0) |  |
| Positive | 387 (30.9) | 199 (31.9) | 188 (29.9) |  |
| Equivocal | 105 (8.4) | 54 (8.7) | 51 (8.1) |  |
| Pathology stage, tumor (T), n (%) |  |  |  | 0.144 |
| T0 or Tis | 42 (3.4) | 22 (3.5) | 20 (3.2) |  |
| T1 | 698 (56.1) | 332 (53.5) | 366 (58.6) |  |
| T2 | 458 (36.8) | 246 (39.7) | 212 (33.9) |  |
| T3 | 42 (3.4) | 19 (3.1) | 23 (3.7) |  |
| T4 | 5 (0.4) | 1 (0.2) | 4 (0.6) |  |
| Pathology stage, nodes (N), n (%) |  |  |  | 0.026 |
| N0 | 686 (54.8) | 341 (54.6) | 345 (54.9) |  |
| N1 | 301 (24.0) | 148 (23.7) | 153 (24.4) |  |
| N2 | 123 (9.8) | 62 (9.9) | 61 (9.7) |  |
| N3 | 142 (11.3) | 73 (11.7) | 69 (11.0) |  |
| Tumor TNM stage, n (%) |  |  |  | 0.077 |
| 0 | 37 (3.0) | 18 (2.9) | 19 (3.0) |  |
| 1 | 449 (36.0) | 212 (34.2) | 237 (37.8) |  |
| 2 | 487 (39.1) | 249 (40.2) | 238 (38.0) |  |
| 3 | 274 (22.0) | 141 (22.7) | 133 (21.2) |  |
| **Intraoperative** |  |  |  |  |
| Surgery type, n (%) |  |  |  | 0.058 |
| Simple mastectomy | 163 (13.0) | 76 (12.2) | 87 (13.8) |  |
| Modified radical | 877 (70.0) | 444 (71.2) | 433 (68.8) |  |
| Wide local excision with node dissection | 141 (11.3) | 68 (10.9) | 73 (11.6) |  |
| Others | 72 (5.7) | 36 (5.8) | 36 (5.7) |  |
| Drugs |  |  |  |  |
| Propofol, mg | 180 [120, 509] | 512 [430, 652] | 120[100, 130] | 3.041 |
| Fentanyl, μg | 150 [100, 200] | 100 [50, 100] | 200 [180, 250] | 2.127 |
| Lidocaine, mg | 20 [0, 40] | 20 [0, 40] | 30 [0, 40] | 0.167 |
| NSAIDS, n (%) | 28 (2.2) | 16 (2.6) | 12 (1.9) | 0.044 |
| **Postoperative treatment** |  |  |  |  |
| Radiotherapy, n (%) | 492 (39.3) | 255 (40.9) | 237 (37.7) | 0.065 |
| Chemotherapy, n (%) | 950 (75.8) | 484 (77.6) | 466 (74.1) | 0.081 |
| Endocrine therapy, n (%) | 821 (65.5) | 409 (65.5) | 412 (65.5) | 0.001 |
| Herceptin, n (%) | 225 (18.0) | 119 (19.1) | 106 (16.9) | 0.058 |
| Recurrence, n (%) | 165 (13.2) | 81 (13.0) | 84 (13.4) | 0.011 |

Notes: Results presented as ‾x ± s or median (P_25_, P_75_) or n (%).

PPA = paravertebral block with propofol general anesthesia; GA = fentanyl with sevoflurane general anesthesia; SMD = standardized mean difference; ASA = American Society of Anesthesiologists; ER = estrogen receptor; PR = progesterone receptor; HER2 = human epidermal growth factor receptor 2.
